# From Tears to Toxins: Mapping Antibiotic Passage Through the Eye–Liver Axis

**DOI:** 10.3390/antibiotics14111069

**Published:** 2025-10-24

**Authors:** Ivan Šoša

**Affiliations:** Department of Anatomy, Faculty of Medicine, University of Rijeka, 51000 Rijeka, Croatia; ivan.sosa@uniri.hr

**Keywords:** ocular surface, liver–eye axis, environmental antibiotic exposure, ocular microbiota, inter-organ signaling, systemic effects

## Abstract

Traditionally used to combat infections, systemic effects of antibiotics are increasingly recognized in the context of absorption through unconventional routes. One such as the ocular surface. This review tackles the bidirectional liver–eye axis, highlighting how trace antibiotic residues from environmental and therapeutic sources affect the tear film, disturb ocular microbiota, and impact liver metabolism. It engages in anatomical pathways, microbial regulation, pharmacokinetics, and systemic immune responses. Additionally, this review discusses forensic uses and new therapeutic strategies, stressing the importance of integrated environmental monitoring and precision medicine to tackle nonmedicinal antibiotic exposure. Due to the absence of results from a systematic literature review, a narrative literature review was undertaken instead. More than 100 studies discussing mechanistic, clinical, and experimental insights were reviewed, with 98 of those studies being documented as source literature. The findings demonstrate that antibiotics may penetrate and be absorbed through the ocular surface, cause modifications of the hepatic first-pass metabolism, and change the activity of cytochrome P450. Correlations were documented between the various liver function biomarkers and the ocular tear film, as well as the thickness of the retinal pigment epithelium. The dysbiosis of eye microbiota may be an indicator of systemic inflammation associated with immune dysregulation. Restoring microbial homeostasis and addressing systemic dysregulation are novel therapeutic approaches, including the use of probiotics, nanoparticle scavengers, and CRISPR. The eye is a sensory organ and a metabolically active organ. Systemically, the eye can affect the liver through the ocular surface and the antibiotics through the liver–eye axis. To protect the systemic health of the individual and the lensed metabolically active eye, the eye and liver must be viewed as a sentinel of systemic balance. Novel therapies will be necessary with the added need for environmental monitoring.

## 1. Methodology of the Literature Review

**Figure 1:**
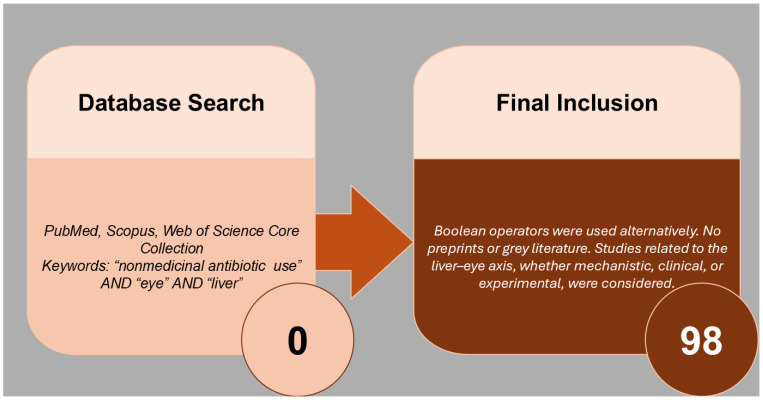
Workflow of the narrative literature review process. This figure outlines the transition from an initially structured systematic review—abandoned due to lack of database results—to a narrative review approach. It illustrates the search strategy, inclusion criteria, and final selection of 98 full-text studies that provided mechanistic, clinical, or experimental insights into the liver–eye axis, ocular microbiota, and systemic effects of nonmedicinal antibiotic exposure.

This literature review was conducted to explore the interrelationship between nonmedicinal antibiotic use, ocular surface changes, and hepatic responses. The methodology was initially conceptualized to follow a structured three-step approach of a systematic review [1]. So, at the very beginning, a comprehensive search was performed across three major databases—PubMed, Scopus, and Web of Science Core Collection—from the inception of each database until 20 August 2025. The keywords “nonmedicinal antibiotic use” AND “eye” AND “liver” were used in combination. As neither of the database searches yielded any results, the systematic review approach was abandoned, and this review was conducted as a less rigid narrative review [2]. Boolean operators were alternatively used to optimize the search and ensure relevance to the liver–eye axis and environmental drug exposure, making the actual “step 2” of the narrative literature review, the process of literature review is presented in Figure 1.

Building on that, this author included papers in English, both primary and secondary publications. Preprints or “grey” literature (information produced by organizations outside of traditional commercial publishing channels, such as government, academia, and business, e.g., Eye Foundation of America) were not considered. For each of the 98 studies finally considered in this manuscript, full-text versions were sought and reviewed. Studies were included if they provided mechanistic insights, clinical correlations, or experimental data relevant to the liver–eye axis, ocular microbiota, or systemic effects of nonmedicinal drug exposure.

## 2. Ocular Surface: A Gateway Beyond Vision

Our eyes and their surfaces are more than just a passive barrier; they are a complex and active connection between the outside world and our internal system. Constantly exposed to air, light, microbes, and chemical agents, it serves as both a sensory platform and a controlling boundary [3,4]. Recent insights reveal that this gentle system of membranes and filaments not only manages local processes in a subcellular manner, but also acts as a pathway for systemic interactions [5,6,7]. In this way, a surprising gateway for non-medicinal substances is created. Accordingly, understanding the permeability and signaling capacity of this gateway opens new perspectives on how ocular exposures can spread throughout the body, influencing distant organs such as the liver [8].

### 2.1. Anatomy and Exposure

**Figure 2:**
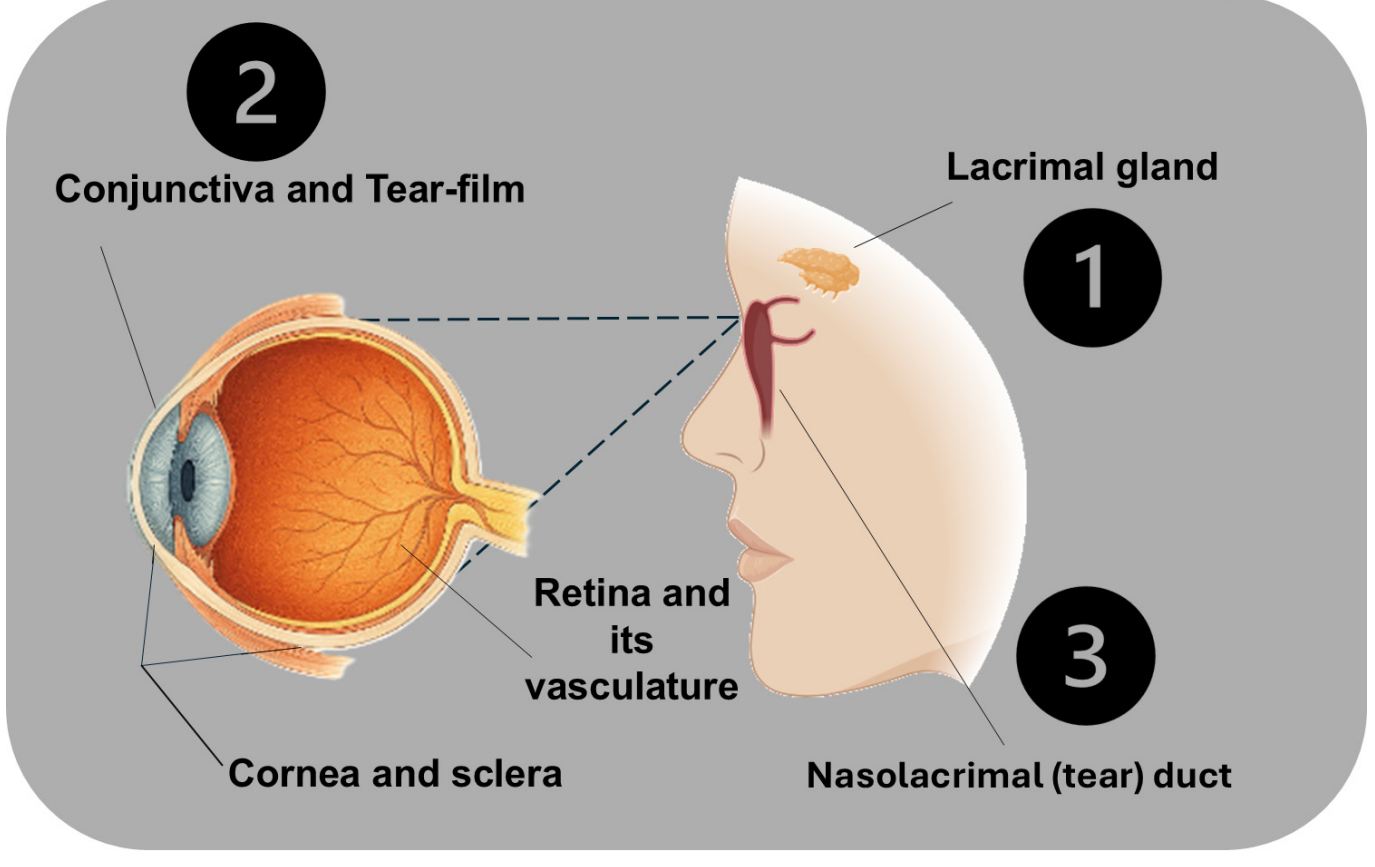
The image provides a representation of the lacrimal system and eye anatomy related to tear production, flow, and drainage. The lacrimal gland (1) is depicted at the upper outer region of the eye socket, which produces tears. Tears then spread over the eye’s surface, including the cornea and sclera, which are labeled clearly in the eye cross-section. The tear film and conjunctiva (2) are shown covering the cornea and sclera, emphasizing the protective layer that keeps the eye moist and free from debris. This part is correctly positioned at the front of the eye anatomy. The nasolacrimal duct (3) is illustrated, running from the inner (nasal) corner of the eye to the nasal cavity, enabling tear drainage.

The surface of our interest is a physical border that includes the corneal epithelium, conjunctival mucosa, tear film, and the nasolacrimal drainage system (Figure 2).

Each component has distinct, specific functions to maintain visual acuity and fortify the protective barrier [9]. The film is even more complex [10,11,12,13]. It contains three layers, lipid, aqueous, and mucin, which serve multiple functions [9,14,15,16]. Moreover, this film provides mechanical protection for the eye enabling uneventful removal of detritus. Also, it contains a wide array of antimicrobial proteins, such as lysozyme and lactoferrin [17]. Its continuous renewal guarantees that environmental factors coexist with the underlying cells, enabling the agents to interact with these factors.

The highly innervated and vascularized conjunctiva provides additional pathways that can serve as routes for absorption, similar to percutaneous absorption [18]. The corneal epithelium has tight junctions that are, to some extent, selective; however, these junctions can be breached by pollutants such as microplastics and irritant chemicals [19]. After these irritants breach the barriers and fluids accumulate, the nasolacrimal duct removes excess fluid containing dissolved solutes. It directs it to the nasal mucosa, from which it then passes into systemic circulation [20,21]. Therefore, these protective and absorptive pathways can be compromised by airborne antibiotics, topical formulations, or particles deposited on the surface of the eye, which can swiftly reach deeper tissues and distant organs, thereby posing a significant health risk [22].

### 2.2. Microbial Modulation

The use of antibiotics can significantly alter the ocular surface microbiota by reducing its diversity and uniformly targeting both helpful and harmful bacteria [23]. Such disruption minimizes the number of protective bacteria that would usually counter opportunistic bacteria, fostering a niche to enable resistant strains to grow [24]. As beneficial microbes decline, key signals that they provide to mucosal immune cells—such as induction of regulatory T cells and production of anti-inflammatory metabolites—are lost. The net effect is a shift toward microbial imbalance, or dysbiosis, that primes the ocular surface for aberrant immune activation [25].

The disruption of the normal microbial balance, called dysbiosis, is increasingly recognized as a critical factor in local disease processes, as well as in systemic conditions [26,27,28]. Even though it is traditionally associated with the gut [29], dysbiosis affects the ocular surface. In this place, a delicate equilibrium of commensal microbes plays a vital role in maintaining immune tolerance and epithelial integrity [30]. Environmental and therapeutic exposure to antibiotics, through unconventional routes such as the ocular surface, can disturb this balance, leading to a cascade of immune dysregulation, epithelial barrier breakdown, and systemic inflammatory signaling [31,32]. In the context of the liver–eye axis, ocular dysbiosis may not only reflect local pathology but also serve as a sentinel marker of broader metabolic and immunological disturbances [33,34,35]. This review focuses on the ocular dysbiosis, examining how antibiotic-driven changes in the eye’s surface microbiota can impact liver function, and vice versa. It redefines the eye as both a target and a source of systemic imbalance.

Use of antibiotics for an extended period of time may change local inflammation due to innate immune receptors [36]. The epithelial and dendritic cells have Toll-like and NOD-like receptors, which can detect microbes [37]. These receptors can become too sensitive and respond vigorously to faint cues when friendly commensal ligands disappear, causing an uncontrollable release of cytokines like interleukins (ILs) -6, -8, and tumor necrosis factor alpha (TNF-α) [38]. This type of inflammation may harm tight junctions, worsen tear-film stability, and fuel continuous epithelial injury alongside immune cell infiltration [5]. In clinical practice, there is a well-documented increase in the expression of dry eyes and refractory redness with the use of topical antibiotics, indicating immune system imbalance [39,40].

Maintaining microbial structure and resilience while strategically limiting antibiotic usage can safeguard ocular health and is the focus of emerging strategies. Reserved use of narrow-spectrum agents or pulse dosing may protect some beneficial flora while more efficiently attacking pathogens. Other therapies including, but not limited to, topical probiotics, prebiotic tear supplements, or bacteriophage cocktails, aim to restore a balanced microbiota and immunological homeostasis [41]. Defining the ideal microbial composition of the ocular surface alongside the creation of microbiota-centric standard protocols for antibiotic treatments are two areas of focus for needed future research.

When it comes to therapies, modulation of the ocular microbiota has emerged as a compelling avenue. Probiotic eye drops containing commensal strains such as *Corynebacterium mastitidis* or *Staphylococcus epidermidis* are being tested to outcompete resistant flora and restore immune homeostasis [42]. These formulations are designed not only to repopulate the ocular surface but also to produce antimicrobial peptides and immunomodulatory metabolites that reinforce epithelial barrier function. Early animal models show that bolstering native microbial communities can dampen proinflammatory cytokine cascades in both conjunctiva and liver [38,43,44], suggesting a bidirectional reinforcement of barrier integrity [45]. Parallel efforts are evaluating topical small-molecule inhibitors of key cytochrome P450 (CYP) isoforms, aimed at preventing hepatic overactivation by absorbed antibiotic residues and thereby normalizing systemic inflammatory tone [46]. These inhibitors, when applied locally, may reduce the hepatic burden of detoxification and limit the production of reactive oxygen species that can damage retinal and choroidal tissues.

### 2.3. Postmortem Ocular Microbiota Shifts

Although environmental and therapeutic factors influence ocular dysbiosis in living individuals [47,48], the microbial ecosystem keeps changing after death, providing forensic clues about the time-dependent spread of microbial succession [49].

A healthy ocular surface is maintained by a sparse but stable community of commensal microbes that outnumber 150-fold fewer bacteria than the adjacent facial skin, reflecting intense immune surveillance and environmental exposure on the eye’s mucosal surface [48,50]. Immediately after death, the collapse of tear-film defenses and epithelial barriers likely precipitates a rapid reshaping of a healthy ocular surface: obligate commensals dwindle as proteolytic and environmental taxa—such as various *Proteobacteria* and spore-formers from the *Bacillaceae* take advantage of this postmortem environment, representing the initial waves of necrobiota colonization [51]. While no detailed surveys have yet been conducted on the ocular necrobiota, similarities with the necrobiota of skin and gut development, again, suggest that these changes constitute an early, and predictable, ocular metamorphosis from life-associated to cadaver-pertaining communities [25,52].

Meanwhile, 2 weeks of oral broad-spectrum antibiotics cause a dramatic depletion of commensal populations and a reduction in overall microbial diversity [24,53], leading to a systemic elevation in lipopolysaccharide levels and resulting in the priming of the eye’s draining.

Beyond increased inflammation, ocular dysbiosis has been directly tied to surface disorders like blepharitis, microbial keratitis, and conjunctivitis, highlighting the protective role of resident microbes in preventing disease [42,54]. In addition, studying conjunctival isolates in a Romanian clinic revealed that cefuroxime, which is commonly used for intracameral prophylaxis (at the end of cataract surgery), showed markedly reduced efficacy. In contrast, agents like vancomycin, aminoglycosides, and fluoroquinolones retained stronger activity [55,56]. Together, these findings underline the importance of combining careful administration of antibiotics with novel strategies—probiotics, microbiota-targeted therapies, or tailored narrow-spectrum treatments—to preserve or restore the delicate microbial balance that safeguards the ocular surface [42].

**Figure 3:**
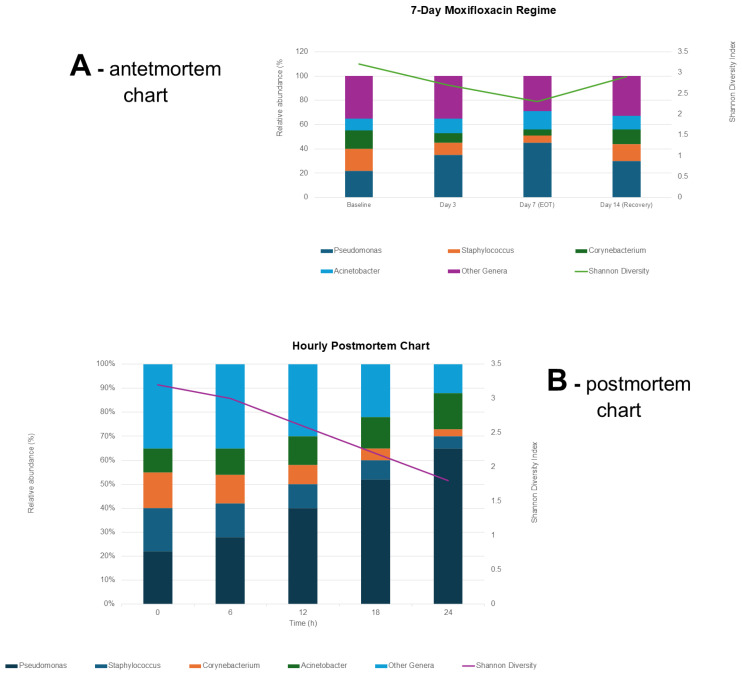
Dynamics of ocular microbiota dysbiosis: (**A**) Temporal changes in microbial composition during and after a 7-day topical moxifloxacin regimen. Stacked bar charts illustrate relative abundance of key genera at baseline, Day 3, Day 7 (end of treatment), and Day 14 (recovery). The accompanying line graph shows a transient reduction in microbial diversity, with partial restoration by Day 14. (**B**) Genus-level shifts in ocular surface microbiota over a 24 h postmortem period, showing increasing dominance of Pseudomonas and declining diversity, as measured using the Shannon index.

Figure 3—panels A, and B show (A) community changes during and after a 7-day topical moxifloxacin regimen. Palate B tracks genus-level shifts in ocular microbiota every six hours after death (0–24 h).

While postmortem changes provide forensic insights, understanding the living bidirectional communication between the eye and liver is crucial for dealing with the systemic implications of ocular antibiotic exposure.

## 3. Liver–Eye Axis: A Metabolic and Immunological Highway

**Figure 4:**
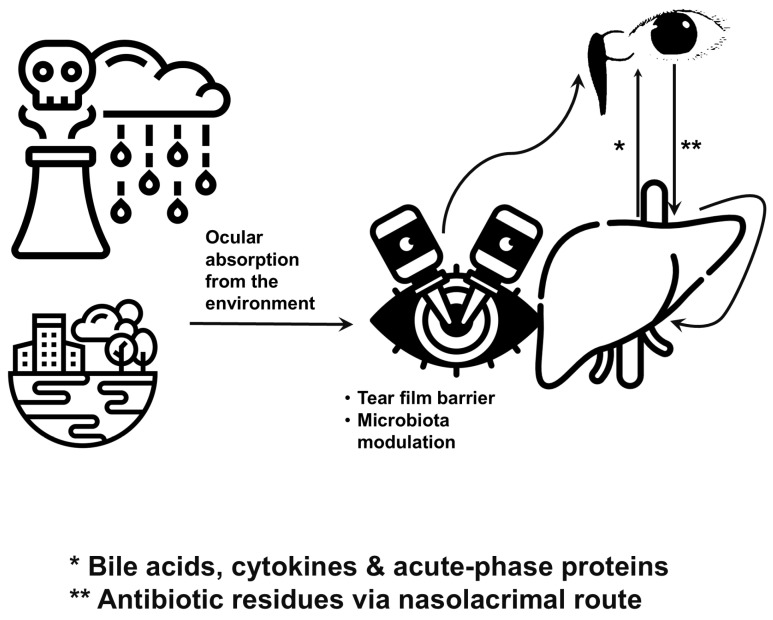
Environmental sources influencing the liver–eye axis through ocular exposure pathways. This figure illustrates how diverse environmental reservoirs—such as airborne antibiotic particles, agricultural runoff, and contaminated consumer products—can deposit trace antibiotics onto the ocular surface. These residues penetrate the tear-film and epithelial barriers, enter systemic circulation via the nasolacrimal duct, and reach the liver, where they modulate metabolic and immunological functions. Hepatic responses, including altered cytokine profiles and bile acid composition, feed back to the eye, reinforcing a bidirectional liver–eye axis. The figure highlights the interconnectedness of environmental exposure, ocular surface microbiota disruption, and systemic health outcomes.

Traditionally, the liver and eye were seen as separate entities, but recent research shows they are part of a complex, two-way network that communicates through metabolic and immune signals (Figure 4). Recent research shows that exposure to certain environmental antibiotics can impact liver function by traveling through the nasolacrimal system, the pathway that allows tears to drain into the nose. Notwithstanding this, the liver plays a role in eye health by controlling the supply of essential nutrients, toxins, and proinflammatory substances [44]. This connection, known as the liver–eye axis, changes our understanding of the eye from just a sensory organ to a crucial part of keeping the body in overall balance. Its understanding may help develop better ways to diagnose and treat eye diseases. This also highlights the importance of taking a comprehensive approach to environmental and medical care [35,56,57,58].

This interconnection is probably best visible during the first pass of vitamin A metabolism. The liver, during that step, takes the dietary provitamin A carotenoid, converts it into retinol esters, and, through retinol-binding protein, supplies the eye with the necessary substrates for the visual process [59,60]. Just alike, drugs administered topically or systemically for ocular conditions may undergo significant biotransformation in the liver, altering their systemic bioavailability and, by extension, their ocular penetration and therapeutic index [61,62].

Beyond metabolism, the liver shapes immune tone through the secretion of acute-phase proteins, complement components, and a host of cytokines that freely circulate to the eye [44,63]. Complement factor production in liver cells is mothing but another testament of the liver-eye connection. Local complement activation in the choriocapillaris and Bruch’s membrane, where abnormal complement turnover has been linked to age-related macular degeneration [64]. Additionally, Kupffer cells and liver-resident lymphocytes modulate systemic levels of interleukins and interferons [65,66]; fluctuations in these mediators can tip the balance between ocular immune homeostasis and pathological inflammation, exacerbating conditions such as uveitis or dry eye disease [40,67].

The eyes reflect pathological liver conditions. What was previously known as nonalcoholic fatty liver disease (NAFLD) is nowadays called metabolic dysfunction-associated steatotic liver disease (MASLD) and appears to be a paragon of this liver-eye connection. This condition is associated with more pronounced retinal microvascular changes and an increased likelihood of diabetic retinopathy, probably due to common pathways involving insulin resistance, oxidative damage, and chronic low-grade inflammation [35,68]. Additionally, cholestatic liver diseases alter the patterns of serum bile acids and desterilize lipid-based tear film, potentially leading to lipid-based evaporative dry eye [40,69,70]. Understanding such relationships is clinically valuable: liver function tests and retinol-binding protein levels may enable the risk stratification of retinal degeneration, and, conversely, liver-patient-emergency frameworks may require modified treatment regimens with ocular medicines due to reduced hepatic clearance limits [71].

Dysbiosis driven by environmental antibiotics disrupts immune homeostasis locally [28]. Epithelial cells deprived of protective microbial signals become hyperresponsive to damage-associated molecular patterns, triggering waves of cytokine release that destabilize the tear-film and compromise barrier integrity [72,73]. Clinically, this manifests as chronic dry eye symptoms, recurrent conjunctivitis, or delayed corneal wound healing [9]. Systemically, the liver’s altered detoxification capacity can exacerbate low-grade inflammation, elevating circulating cytokines that further prime ocular tissues for injury and impair vascular health in the retina [38]

### 3.1. Correlations Through the Lens of Ocular Surface Microbiota

A hypothetical dataset (Supplementary Material File S1) illustrating how combined liver metabolic biomarkers, immunological mediators, and retinal structural measurements can be aligned to explore the liver–eye axis concept was designed using Microsoft Copilot (Microsoft Corporation. (2024). Microsoft Copilot (Version 1.0) [AI language model]. from https://copilot.microsoft.com, accessed on 3 September 2025). A hypothetical model was considered, utilizing seven liver function markers used in a large-scale cohort study (ALT, AST, GGT, ALP, TBIL, TP, ALB). Complement C3 and CRP represent key immune-inflammatory mediators described in the metabolic–immunological crosstalk of the liver–eye axis. In addition, the model considered retinal pigment epithelium (RPE) and inner/outer photoreceptor segment–optical coherence tomography (OCT) measured RPE (ISOSRPE) thicknesses and tear-film thickness to capture ocular structural changes linked to liver function. Table presents Pearson correlation coefficients between systemic biomarkers, immunological mediators, tear-film thickness, and retinal pigment epithelium (RPE) thickness (Table 1.).

Liver enzymes, including ALT, AST, GGT, ALP, and TBIL, each correlate very highly with both tear-film and RPE thickness. This implies that elevations in liver function markers tend to coincide with thicker tear films and RPE layers. Immunologic markers mirror hepatic trends; what can be seen as complement C3 and CRP correlate positively (≈0.95–0.98) with ocular parameters. Correlation suggests that low-grade systemic inflammation covaries with changes in tear-film stability and retinal structure. Tear-film and RPE are strongly interlinked, as tear-film thickness correlates at 0.981 with RPE thickness. And this indicates a tight relationship, consistent with the liver–eye axis concept. Altered hepatic function and inflammatory tone may drive fluid dynamics in the tear-film and structural remodeling of the RPE. Conversely, ocular surface disturbances (e.g., dysbiosis or subclinical inflammation) could feed back to influence liver metabolism and systemic immune markers.

Inverse relationship (strong negative correlations) of serum proteins (TP and ALB) with both ocular measures (−0.94 and −0.97) indicates that higher circulating protein levels might associate with thinner tear films and RPE [74].

- Positive correlations of ALT, AST, GGT, ALP, TBIL, Complement C3, and CRP with tear-film thickness imply that systemic inflammation or mild hepatic injury coincides with tear-film expansion [75].
- A thicker tear-film can dilute antimicrobial peptides and disrupt nutrient gradients that normally sustain commensal bacteria, fostering dysbiosis [42].
- Conversely, the strong negative association with TP and ALB suggests lower tear-film protein content—key substrates for resident microbes—when serum proteins are elevated [74,76].

The extremely high correlation between tear-film and RPE thickness (0.981) indicates that surface perturbations (e.g., altered microbiota or tear osmolarity) feed back to the posterior segment [77].

- Subclinical inflammation on the ocular surface can release cytokines that traverse to the retina, triggering glial activation and subtle RPE hypertrophy, which in turn may further disturb immune surveillance at the conjunctiva [78].
- Elevated liver enzymes and acute-phase proteins often accompany antibiotic residues that have traversed the nasolacrimal system—antibiotics themselves are potent modulators of microbial diversity [79].
- Complement C3’s positive correlation underscores an immune-driven culling of certain bacterial taxa, while CRP’s similar trend highlights low-grade inflammation that may perpetuate microbial dysbiosis and contribute to systemic immune modulation [80].

These modeled correlations lay the foundation for practical applications of the l-eye axis in toxicology, diagnostics, and forensic science.

### 3.2. Forensic and Clinical Implications for Ocular Surface Microbiota

The ocular surface harbors a diverse bacterial microbiota, with a few fungi and viruses. *Corynebacterium*, *Staphylococcus*, and *Cutibacterium*, have been identified in recent culture-independent and genomic analyses as core residents who also challenge the notion of ocular sterility [47,81]. This casts a new light onto our understanding of the microbiome’s role in maintaining ocular health [50].

Researchers have found that changes in the eye’s microbial communities, known as dysbiosis, are linked to conditions like keratitis, blepharitis, conjunctivitis, and dry eye syndromes [82]. This has led to the development of diagnostic tools and targeted treatments based on the microbiome (Table 2). In the field of forensics, the unique makeup and changing patterns of eye microbiota are starting to be used as possible markers for identifying individuals and estimating the time of death [57].

As we advance, incorporating rapid sequencing platforms into ophthalmic clinics could allow for real-time microbiota profiling, enabling personalized treatments [84]. In addition, forensic applications might use machine-learning algorithms to match ocular microbial signatures with suspects or reconstruct time-since-death profiles [85,86].

1. Biomarker-Guided Microbiome Profiling: In cases of unexplained ocular surface disease, pairing tear-film thickness/OCT-derived RPE measures with 16S rRNA sequencing can pinpoint dysbiotic signatures.
2. Nonmedicinal Antibiotic Surveillance: Trace antibiotic detection in tears, together with elevated liver enzymes, flags environmental exposures that may covertly reshape the ocular microbiome and compromise barrier integrity.
3. Therapeutic Monitoring: Restoration of a healthy microbiota (via probiotics or tailored antibiotic stewardship) should normalize both tear metrics and RPE thickness, serving as quantifiable readouts of microbial resilience [27,42].

## 4. Antibiotics and Eye Microbiota

Antibiotics can leave measurable traces both in systemic biomarkers (especially of liver function) and in ocular compartments [26,87,88]. Given the strong correlations observed between liver enzymes, inflammatory markers, tear-film composition, and retinal pigment epithelium (RPE) thickness [89,90], antibiotic exposures may now be a crucial factor driving changes in these parameters, opening up new opportunities for forensic toxicology. Table 3 highlights the tear-film kinetics and liver functions of five frequently used antibiotics, illustrating their pharmacokinetic behavior and systemic effects via the liver–eye axis.

Antibiotic exposure, whether topical or systemic, can substantially disturb the ocular surface microbiota, resulting in dysbiosis—a condition characterized by microbial imbalance. Not only does this disruption reduce the diversity of commensal organisms supporting immune homeostasis and epithelial integrity, but it also impairs signaling to mucosal immune cells. This leads to immune imbalance, with overactive innate receptors and high cytokine levels, which weaken tear-film stability and damage the epithelium. In clinical sense, antibiotic-induced dysbiosis is associated with dry eye syndrome, blepharitis, microbial keratitis, and increased susceptibility to opportunistic infections [57,72,91]. Prolonged use or use of broad-spectrum antibiotics may also lead to resistant strains, further complicating treatment [92].

Tear-film can track water-soluble and lipophilic antibiotics delivered via topical instillation, environmental drift, or systemic circulation [22]. Concentrations of these antibiotics in ocular fluids and tissues often mirror plasma levels and can persist even when blood samples are degraded or unavailable [11,83]. Tissue penetration studies (e.g., moxifloxacin) show that higher ALT and AST levels predict lower antibiotic diffusion into interstitial spaces (but note that there is no direct correlation between ALT and AST levels and antibiotic diffusion [79])—including ocular tissues—due to altered protein binding and vascular permeability [93]. These findings highlight the importance of careful antibiotic use and the development of strategies that preserve microbiota, such as narrow-spectrum agents, pulse dosing, and topical probiotics, to sustain ocular microbial resilience.

Beyond therapeutic use, environmental exposure to antibiotics presents additional challenges to ocular microbiota and systemic health.

## 5. Nonmedicinal Antibiotic Use: Mechanisms and Implications

The continued high levels of global antibiotic use pose a significant public health challenge. Between 2016 and 2023, it grew by 16.3%, going from 29.5 billion to 34.3 billion defined daily doses (DDDs). This represents the growth in overall consumption from 13.7 to 15.2 DDDs per 1000 inhabitants per day. Upper-middle- and lower-middle-income countries saw the most significant increases, while high-income countries experienced temporary declines during the COVID-19 pandemic. Projections indicate that global antibiotic use could grow by more than 50% by 2030, reaching 75.1 billion DDDs if no targeted measures are implemented [94]. Such trends draw attention to the urgency of rational antibiotic use and the need to monitor non-traditional exposure routes, such as the ocular surface, which may contribute to systemic effects and antimicrobial resistance [95,96].

Nonmedicinal antibiotic exposure occurs when trace amounts of antibiotics—originating from environmental runoff, aerosolized particles, or contaminated consumer products—contact the ocular surface [97]. Unlike deliberate ophthalmic administration, these low-dose, chronic exposures are unpredictable in timing and concentration. On the eye, deposited antibiotic molecules dissolve into the tear-film layers, where they can persist long enough to interact with epithelial and immune cells [91]. Because the tear-film is continuously renewed and drains through the nasolacrimal duct, these compounds gain access to systemic circulation without first-pass hepatic metabolism, creating a direct conduit between environmental reservoirs and internal organs.

After crossing the ocular epithelium, antibiotics enter conjunctival capillaries and lymphatic channels [12,13,83]. Even subtherapeutic concentrations can exert selective pressure on the resident microbiota, reducing commensal populations and encouraging the outgrowth of resistant or opportunistic strains [98]. At the same time, antibiotics bind to plasma proteins and distribute to peripheral tissues, including the liver. In hepatocytes, they may inhibit or induce CYP enzymes [99], alter phase II conjugation pathways, and modulate expression of drug-transporters [100,101]. These hepatic changes affect back to the eye via altered levels of bile acids, acute-phase proteins, and inflammatory mediators, reinforcing the concept of a liver–eye axis [70]

**Figure 5:**
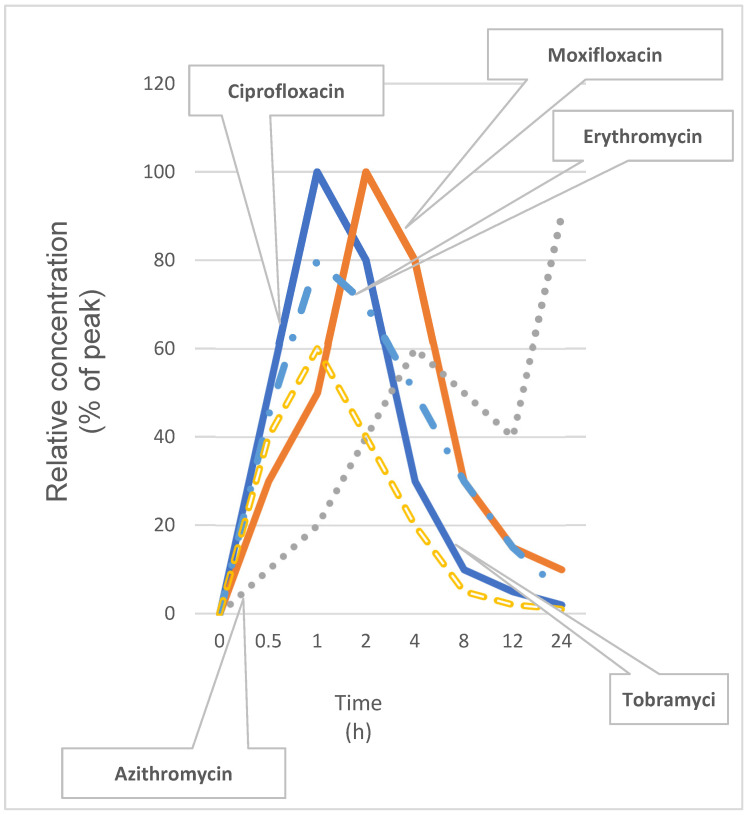
Ocular surface pharmacokinetics of selected topical antibiotics. Tear-film concentration–time profiles over 24 h for ciprofloxacin, moxifloxacin, azithromycin, tobramycin, and erythromycin following a single topical dose. Curves show rapid absorption and elimination kinetics for fluoroquinolones and macrolides, with azithromycin exhibiting prolonged retention.

The implications of this exposure extend beyond ocular morbidity. Persistent antibiotic residues in liver cells can impair mitochondrial function and provoke oxidative stress, laying the groundwork for fatty liver changes or cholestatic injury. Over time, subclinical hepatic dysfunction may erode retinal pigment epithelium health, accelerate microvascular damage, and heighten susceptibility to conditions such as nonproliferative retinopathy. Recognizing these mechanisms underscores the need for environmental antibiotic monitoring, stricter wastewater controls, and novel ocular barrier enhancers that can intercept unwanted molecules before they trigger systemic ripple effects [102,103]. To visualize the pharmacokinetic behavior of selected topical antibiotics. Figure 5 presents tear-film concentration–time profiles over 24 h, highlighting differences in absorption and retention among fluoroquinolones and macrolides. Table 4 complements the pharmacokinetic profiles by summarizing T_max_, half-life, and documented hepatic interactions for each antibiotic, thereby reinforcing their roles in liver–eye axis signaling.

## 6. Emerging Research and Therapeutic Potential

Recent studies are uncovering subtle methods to reduce the unintended effects of environmental antibiotic residues on the eye and its connection with the liver. Advanced research is looking into nanoparticle carriers that can target and neutralize antibiotic molecules in the tear-film before they spread into eye tissues. These carriers, typically made of biocompatible polymers or lipid-based vesicles, are designed to recognize specific antibiotic structures and trap them without triggering immune reactions. By intercepting these molecules at the ocular surface, researchers aim to prevent downstream effects such as microbial dysbiosis, epithelial inflammation, and systemic absorption. Concurrently, investigators are leveraging high-resolution mass spectrometry and metabolomic profiling to map the fate of micropollutants in tears and trace their metabolic byproducts in hepatocytes. These tools are illuminating previously unrecognized detox pathways, including alternative conjugation routes and compensatory enzyme networks that activate under chronic low-dose exposure. Such analytical advances promise to refine our understanding of dosage thresholds that trigger dysbiosis or hepatic enzyme perturbation, laying the groundwork for precision environmental ophthalmology [104]. Accordingly, Figure 4 illustrates the route of environmental pollutants alongside the liver-eye axis, and bidirectional communication between the eye and liver [45]. Visualization emphasizes the role of tear-film barrier integrity and microbiota modulation in ocular–systemic interactions. Furthermore, this diagram highlights how antibiotic residues may reach the liver via the nasolacrimal route, while hepatic-derived bile acids, cytokines, and acute-phase proteins can influence ocular surface immunity [70].

Advances in technologies like gene editing are moving forward quickly. Methods based on clustered regularly interspaced short palindromic repeats (CRISPR) are explicitly designed to target resistance genes right on the eye’s surface. Researchers use bioadhesive gels or mucoadhesive nanoparticles to deliver RNA [105,106], to eliminate resistant bacteria while selectively preserving beneficial resident microbes. This strategy offers a precise solution to the growing problem of ocular antibiotic resistance, particularly in post-surgical or chronic dry eye cases [107,108]. Additionally, RNA interference platforms are being customized to block hepatic transporters that move antibiotic temporarily conjugates back into circulation, providing a reversible way to disrupt harmful liver–eye feedback loops [109,110]. These molecular strategies have the potential to deliver targeted therapies that improve both local eye health and systemic metabolic function, possibly revolutionizing how we treat environmentally driven ocular diseases [111].

Next-generation research may include the mapping of bidirectional liver-eye communication via extracellular vesicles and microRNA, as well as the circadian alignment of hepatic metabolism with retinal clock genes [45]. Tailored omega-3s and controlled vitamin A dosing may target liver health and preserve ocular and hepatic function. Eventually, eye and liver nurturing may be achieved through vision-dependent frameworks and the deciphering of this highway of metabolism and immunology.

Looking ahead, multidisciplinary collaborations will be key to translating these discoveries into clinical practice. Integrating environmental monitoring with patient-specific risk profiling could enable early identification of individuals most vulnerable to low-grade antibiotic exposure. Advanced biomarkers—such as tear-film proteomic signatures, hepatic microRNA panels, or conjunctival transcriptomic shifts—may soon guide personalized treatments, from barrier-strengthening lubricants to targeted microbial therapies [90,112]. Regulatory frameworks must adapt to these new modalities, particularly as they increasingly blur the boundaries between ophthalmology, toxicology, and precision medicine [113]. Environmental sciences converge with microbiology and molecular therapeutics, offering a path toward safeguarding ocular integrity while preserving the delicate equilibrium of molecules alongside the liver–eye axis.

### 6.1. CRISPR-Based Approaches

These gene-editing tools enable precise targeting of antibiotic resistance genes on the ocular surface, so it can be confidently stated that CRISPR/Cas systems are a transformative technology for eye health [105,114]. This technology provides a way to reduce resistant strains while maintaining beneficial microbiota. It could be used in many applications, such as post-surgical infections, chronic dry eye disease, and ocular surface disorders related to dysbiosis [42]. As advances are made in pharmaceuticals, progress in CRISPR-based methods is likely to be matched by improvements in delivery platforms [115]. Future integration of CRISPR-based approaches with RNA interference and microbiome engineering for synergistic effects should still be pursued, while also considering off-target effects, immune reactions, and regulatory challenges for clinical use. This is especially relevant given the potential role of CRISPR-based strategies in technologies like bioadhesive gels and mucoadhesive nanoparticles that ensure localized action without systemic exposure [105].

### 6.2. Nanoparticle-Based Scavengers

Nanoparticles designed from biocompatible polymers or lipid vesicles can neutralize antibiotic residues in the tear film before systemic absorption. These carriers may either bind selectively to antibiotic molecules, prevent downstream effects such as microbial imbalance and hepatic enzyme perturbation, or offer potential for environmental ophthalmology by intercepting pollutants at the ocular frontier [116].

### 6.3. Probiotic and Microbiota-Centric Therapies

Topical probiotics containing commensal strains aim to restore microbial diversity. This should produce antimicrobial peptides and immunomodulatory metabolites, reducing proinflammatory cytokine cascades locally and systemically [117].

### 6.4. RNA Interference Platforms

RNAi-based therapeutics are being explored to block hepatic transporters that recycle antibiotic conjugates (which is highly topical in the context of this review) [109,118]. RNA interference platforms interrupt harmful liver–eye feedback loops [119]. Furthermore, these provide reversible modulation of systemic inflammatory tone [120,121].

## 7. Conclusions

The ocular surface constitutes a dynamic interface through which environmental antibiotics can bypass conventional pharmacokinetic barriers, disseminating into systemic circulation and engaging hepatic metabolic and immunologic networks. Our review highlights how trace antibiotic residues disrupt the ocular surface microbiota, compromise barrier integrity, and amplify low-grade inflammation both locally and in the liver [23,50].

Evidence in this review shows that long-term exposure to low levels of chemicals from the environment, farming, or everyday products can disrupt normal bacteria and overactivate the immune system [6,40,97,102]. The subsequent disturbances in CYP activity [99], bile acid composition [70], and acute-phase protein synthesis orchestrate a bidirectional liver–eye axis that may accelerate retinal microvascular damage and surface pathologies.

The bidirectional nature of the liver–eye axis also opens new avenues for diagnostics and forensic applications. Tear-film biomarkers, microbial succession patterns, and postmortem shifts in ocular microbiota may serve as indicators of systemic exposure, immune status, or time of death. These insights call for integrated environmental monitoring and precision medicine frameworks that consider the eye and liver as co-regulators of systemic equilibrium.

Innovative strategies—including nanoparticle-based scavengers, microbiota-tailored probiotics, targeted enzyme inhibitors, and CRISPR-derived gene-editing platforms—offer promising avenues to intercept antibiotic pollutants at the ocular frontier and restore homeostatic feedback loops [105,122]. By enabling precise editing of microbial genomes or host immune regulators, CRISPR tools may allow for targeted restoration of microbial balance and modulation of inflammatory pathways. Early-stage models suggest that CRISPR can selectively eliminate resistant ocular pathogens or reprogram epithelial responses, potentially reversing dysbiosis and mitigating hepatic overactivation. Simultaneously developing high-resolution exposure maps and multimodal diagnostic biomarkers will be essential to customize interventions based on individual exposure profiles and microbiota settings, helping to advance clinical options [98,123,124].

As we advance, it is increasingly becoming essential to integrate environmental surveillance, precision ophthalmology, toxicogenomics, and personalized medicine, all driven by structured risk assessment frameworks, to shape regulatory policies [125]. Therapeutic paradigms that safeguard ocular integrity while mitigating systemic effects. By reframing the eye as both a watch and a pathway for internal metabolic flow, we can foster interdisciplinary solutions to address the increasing challenge of nonmedicinal antibiotic exposure [113].

## Figures and Tables

**Table 1:** Correlation coefficients of systemic and ocular biomarkers with tear-film thickness and retinal pigment epithelium (RPE) thickness: data from the hypothetical model.

Parameter	Correlation Coefficient with Tear-Film Thickness	Correlation Coefficient with Retinal Pigment Epithelium (RPE) Thickness	Unit
ALT	0.96	0.99	U/L
AST	0.96	0.99	U/L
GGT	0.96	0.99	U/L
ALP	0.96	0.99	U/L
TBIL	0.96	0.99	mg/dL
TP	−0.94	−0.97	g/L
ALB	−0.94	−0.97	g/L
Complement C3	0.95	0.98	g/L
CRP	0.95	0.98	mg/L
ISOSRPE thickness	−0.93	−0.95	µm
RPE thickness	0.98		µm
Tear-film thickness		0.981	µm

Abbreviations: ALT: alanine aminotransferase; AST: aspartate aminotransferase; GGT: gamma-glutamyl transferase; ALP: alkaline phosphatase; TBIL: total bilirubin; TP: total protein; Data are synthetic and illustrative. Confidence intervals are two-sided 95% bootstrap percentile intervals. The table is illustrative and not derived from observed patient data; interpret it as conceptual support for hypotheses to be tested in real cohorts.

**Table 2:** Forensic applications of ocular markers within the liver–eye axis framework.

	Contents/Data Type	Why this Column Matters	Example Entry
Application	Short name of the forensic/clinical use (text).	Quickly orients reader to the use case.	Detection of trace antibiotics.
Rationale	One-sentence biological or analytical justification (text).	Links application to mechanism or evidence.	Tear-film retains drugs when blood is degraded.
Sample/Matrix	Which specimen to collect (controlled vocabulary).	Guides sampling choice and downstream methods.	Tear film; conjunctival swab; ocular tissue.
Analytical method	Primary assay(s) and platform (text).	Specifies how the marker will be measured.	LC-MS/MS; 16S rRNA sequencing; OCT.
Typical detection window/stability	Timeframe and conditions (text + units).	Sets realistic expectations for when marker is informative.	0–7 days post-exposure; stable at −20 °C.
Sensitivity/LOD	Typical limit of detection or sensitivity range (numeric or “unknown”).	Technical feasibility and comparability across labs.	ng/mL range (0.5–5 ng/mL).
Evidence level	Tiered scale (Preclinical/Observational/Validated/Speculative).	Conveys maturity and confidence for application.	Observational human data.
Sampling notes	Practical collection volume, technique, preservative (text).	Reduces preanalytical variability and contamination.	Capillary tear collection 5–10 µL; freeze in methanol.
Main limitations/caveats	Key confounders, stability issues, forensic/legal limits (text).	Prevents overinterpretation and informs study design.	Environmental contamination; postmortem degradation.
Forensic validity/PMI use	Yes/No/Partial + applicable PMI range (text).	Directly informs forensic utility and limitations.	Partial—informative 0–72 h under cool conditions.
Suggested QC/controls	Recommended quality controls or reference standards (text).	Ensures analytical reliability and reproducibility.	Spiked tear standards; blank swab controls.
References	Key supporting citations (numbered).	Directs the reader to evidence and methods.	[11,22,83]

Abbreviations: LC MS/MS—Liquid chromatography–tandem mass spectrometry; 16S rRNA16S ribosomal RNA (gene sequencing for bacterial profiling); OCT Optical coherence tomography; LOD—Limit of detection; QC—Quality control; PMI—Postmortem interval; ng/mL—Nanograms per millilitre; µL—Microlitre; °C—Degree Celsius; LC MS—Liquid chromatography–mass spectrometry; PCR—Polymerase chain reaction; NGS—Next generation sequencing; RPE—Retinal pigment epithelium; LFTs—Liver function tests; CV—Coefficient of variation.

**Table 3:** Ocular surface pharmacokinetics and liver–eye axis roles of selected antibiotics.

Antibiotic	Ocular Surface Kinetics	Role in Liver–Eye Axis
Ciprofloxacin	Peak tear concentration within 1 h; half-life ~1.5 h.	Inhibits CYP1A2 in hepatocytes; shifts hepatic detox pathways and modulates ocular inflammatory tone.
Moxifloxacin	Peak in tears at ~2 h; half-life ~2.5 h; high corneal penetration.	Alters hepatic oxidative stress pathways; influences retinal immune cell activation.
Azithromycin	Prolonged tear-film residency (~24 h); strong tissue binding.	Activates Kupffer cells indirectly; reduces systemic IL-6 levels and reshapes ocular immune responses.
Tobramycin	Rapid absorption; half-life ~1.8 h; minimal systemic uptake.	Limited hepatic metabolism; low direct liver–eye signaling but may drive selection of resistant flora.
Erythromycin	Peak in ~1.5 h; half-life ~2 h; accumulates in conjunctiva.	Inhibits bile acid transporters; can provoke mild cholestasis and exacerbate ocular surface inflammation.

Abbreviations: h: hour; t½: half-life; CYP: cytochrome P450; IL-6: interleukin-6.

**Table 4:** Summary table of pharmacokinetic parameters (T_max, t_½) alongside documented hepatic interactions—such as CYP modulation, oxidative-stress effects, and cytokine changes—linking each antibiotic to downstream liver–eye signaling.

Antibiotic	Tmax (h)	t½ (h)	Key Liver–Eye Axis Role
Ciprofloxacin	1	1.5	Inhibits CYP1A2; modulates ocular inflammatory tone
Moxifloxacin	2	2.5	Alters hepatic oxidative stress; influences retinal immune activation
Azithromycin	24	24	Activates Kupffer cells; reduces systemic IL-6
Tobramycin	1	1.8	Minimal hepatic metabolism; may drive selection of resistant flora
Erythromycin	1.5	2	Inhibits bile acid transporters; can exacerbate ocular inflammation

Abbreviations: T_max—Time to Maximum Concentration; t½—Half-life; CYP—Cytochrome P450; IL-6—Interleukin-6.

## Data Availability

Available on request.

## Supplementary Materials

The following supporting information can be downloaded at: https://www.mdpi.com/article/10.3390/antibiotics14111069/s1, and are available online at https://uniri-my.sharepoint.com/:x:/g/personal/ivan_sosa_uniri_hr/EVfru9tyPhBNgLBsA9HGKooBqZsdoQdIM5sCrcLjlu-tDw?e=l6uU52 (accessed on 22 October 2025): File S1. This file contains four sheets with detailed data supporting the findings of the manuscript. Hourly Postmortem Line Chart: Microbial abundance and Shannon diversity index measured at 6 h intervals postmortem, including genera such as *Pseudomonas*, *Staphylococcus*, *Corynebacterium*, *Acinetobacter*, and others. 7-Day Moxifloxacin Regime: Microbial composition and diversity at baseline, Day 3, Day 7 (end of treatment), and Day 14 (recovery), highlighting the impact of moxifloxacin on ocular microbiota. Concentration–Time Data for Lin: Pharmacokinetic profiles of five antibiotics (*Ciprofloxacin*, *Moxifloxacin*, *Azithromycin*, *Tobramycin*, and *Erythromycin*) over a 24 h period. Inset Summary Table Data: Key pharmacokinetic parameters (Tmax, t½) and liver–eye axis roles for the five antibiotics studied, including mechanisms such as CYP inhibition, Kupffer cell activation, and bile acid transporter modulation.

## Abbreviations

The following abbreviations are used in this manuscript:

Abbreviation	Full Term/Meaning
16S rRNA	16S ribosomal RNA
ALB	Albumin
ALP	Alkaline phosphatase
ALT	Alanine aminotransferase
AMR	Antimicrobial resistance
ANOVA	Analysis of variance
AST	Aspartate aminotransferase
CV	Coefficient of variation
CRP	C-reactive protein
CYP	Cytochrome P450
GGT	Gamma-glutamyl transferase
DDD	defined daily dose
IL6	Interleukin-6
ISOSRPE	Inner/outer segment—retinal pigment epithelium thickness
LOD	Limit of detection
LCMS	Liquid chromatography–mass spectrometry
LCMS/MS	Liquid chromatography–tandem mass spectrometry
LFTs	Liver function tests
LPS	Lipopolysaccharide
MASLD	Metabolic dysfunction-associated steatotic liver disease
NAFLD	Nonalcoholic fatty liver disease
NGS	Next-generation sequencing
OCT	Optical coherence tomography
PCR	Polymerase chain reaction
PMI	Postmortem interval
QC	Quality control
RPE	Retinal pigment epithelium
RT	Room temperature
t½	Half-life
T_max	Time to maximum concentration
TBIL	Total bilirubin
TP	Total protein
µL	Microlitre
°C	Degree Celsius
